# Younger Stroke Survivors' Experiences of Family Life in a Long-Term Perspective: A Narrative Hermeneutic Phenomenological Study

**DOI:** 10.1155/2012/948791

**Published:** 2012-12-11

**Authors:** Randi Martinsen, Marit Kirkevold, Unni Sveen

**Affiliations:** ^1^Department of Nursing Science, Faculty of Medicine, Institute of Health and Society, University of Oslo, P.O. Box 1130, Blindern, 0318 Oslo, Norway; ^2^Department of Nursing and Mental Health, Hedmark University College, P.O. Box 400, 2418 Elverum, Norway; ^3^Department of Physical Medicine and Rehabilitation, Oslo University Hospital, P.O. Box 4950, Nydalen, 0424 Oslo, Norway; ^4^Faculty of Health Science, Oslo and Akershus University College of Applied Sciences, P.O. Box 4, St. Olavs plass, 0130 Oslo, Norway

## Abstract

The psychosocial consequences following a stroke are known to be challenging, influencing the stroke survivors' ability to participate in and carry out the taken-for-granted roles and activities in family life. This study explored how living with the consequences of stroke impacted on family life in the late recovery phase, that is, six months or more after stroke onset. Twenty-two stroke survivors aged 20–61 years were interviewed in-depth six months to nine years after stroke onset. The interviews were analyzed applying a narrative, hermeneutic phenomenological approach. The findings revealed challenges that varied with time, from an initial struggle to suffice in and balance the relationships and roles within the family early after the stroke, towards a more resigned attitude later on in the stroke trajectory. The struggles are summarized in two main themes: “struggling to reenter the family” and “screaming for acceptance.” Nonestablished people living with stroke and stroke survivors in parental roles seem to be particularly vulnerable. Being provided with opportunities to narrate their experiences to interested and qualified persons outside the home context might be helpful to prevent psychosocial problems.

## 1. Introduction

A number of studies suggest that suffering from a stroke at a young age (i.e., younger than 67 years old) may impact significantly on family relationships and functioning [1–9]. This is related to the fact that a stroke usually leads to long-lasting consequences that influence the stroke survivor's abilities to carry out family roles and engage in family relations in the same manner as before the stroke [2, 9, 10]. Among young stroke survivors, these consequences are frequently related to psychological challenges, such as loss of control and self-efficacy, dependency, feelings of isolation, or loss of self as previously known [3, 11–14]. In addition, invisible symptoms, such as memory loss, concentration problems, fatigue, and neglect, may be particularly challenging and frustrating, not only to the young stroke survivors, but to their families as well [1, 2, 15]. 

 Today's families vary greatly in composition, functioning, and the roles and responsibilities they are expected to address, depending on the cultural and social context. Family processes and family roles are influenced by a number of factors, including genetic heritage, behaviours, attitudes, and expectations as well as developmental life cycle transitions, unpredictable factors, and historical events [16, 17]. 

Despite a great variation in family patterns, the family life cycle seems to have some universal characteristics, at least within western cultures [16, 17]. During the initial adult phase, the single young adult period, the focus is on independence and emancipation as the young adult separates from the family of origin by leaving home and developing intimate peer relationships. Personal goals such as establishing one's identity, assuming emotional and financial responsibility, working, studying, and committing to a career path dominate the young adult's life. Thus, turning to parents or other family members for advice may be challenging [16, 18]. Nevertheless,  following a stroke, the younger stroke survivors have been found to increase dependency on their parents [19].

The midphase of adult life is most often focused on forming a partnership, getting married, and becoming parents. Life tasks such as raising children, performing household tasks, launching a career, and engaging in financial commitments are comprehensive and long lasting. Individuals in this phase form new bonds, must face their own children's independence, and later address their parents' frailty [16]. The negotiating of different roles during this phase is traditionally viewed as one of the most difficult transitions [18]. Studies point to altered relationships between stroke survivors and their partners during this phase [2, 6, 9, 12, 19–21]. Even a minor stroke may negatively impact marital relationships [22] and possibly result in disrupted marriages [5, 9, 19]. However, younger marriages seem to suffer more from broken relationships than do long-lasting relationships because of the ability of those in longer-lasting marriages to rearrange and adjust to the changes brought about after a stroke [9, 19]. 

The parent role is psychologically bonding, influencing the social balance between work, friends, siblings, and parents [16]; thus, adapting the parental role after a stroke has been found to be challenging [1, 4, 7, 9, 19]. Being separated from children over time was found to have a negative impact, particularly for single mothers [9]. Female stroke survivors have expressed concern about their roles as mothers and housewives [1], while both women and men worry about the economic situation of the family [1, 23]. Studies have also underlined that men's quality of life was negatively influenced when they experienced shortcomings in terms of being able to protect their family [20] or when living alone [23].

Parents at midlife develop adult relationships with their grown children, in-laws and grandchildren, while also dealing with the disabilities and death of their aging parents. During this “empty nest” period, children home leaving requires fewer parenting commitments and, to most, results in a more fortunate economic situation for the parents. To some extent, this phase is a welcome and positive one, allowing the parents to explore new arenas and new roles; others, however, find this new phase of their lives to be empty and meaningless [16].

Although friendships tend to be more temporal than family ties [24], friends, neighbours, and extended family may often serve as a resource to the immediate family during both positive and troubled times [16]. Social interactions within and outside the immediate family are considered critical for those trying to reintegrate into the community [4, 5, 12]. Friendships are often affected [1, 10, 15, 21], and the loss of friends can be a traumatic experience [21], while the opportunity to establish both new intimate relationships and new friendships has been found to be quite limited after an acquired brain injury [15, 21].

## 2. Aim and Research Question 

Despite the existing knowledge of how the long-lasting impacts challenge the daily lives of stroke survivors under the age of 67 years [2, 10, 15, 25], in-depth research on how a stroke impacts on the family life of the stroke survivor over an extended period of time is still limited. Knowing that family life is a process that may be filled with various challenges [16–18], it is pertinent to explore how living with a stroke influences family life during the young adult phase, the mid-phase, and the “empty nest” phase, respectively, from the stroke survivors' point of view. Such knowledge will make it easier to provide adequate support and guidance following a stroke. Consequently, the aim of this study was to gain a deeper understanding of how a stroke impacts on family life six months or more after the stroke, as experienced by young stroke survivors aged between 18 and 67 years. The following research question was asked: how do stroke survivors aged between 18 and 67 years experience their roles and interactions within the family six months or more after suffering a stroke?

## 3. Research Methodology

The study had an exploratory design and applied a qualitative, hermeneutic phenomenological approach. The hermeneutic phenomenological method is appropriate to understand the meaning of lived experiences [26, 27] and was therefore chosen to explore younger stroke survivors' experiences of family life. 

### 3.1. Sample

This study was carried out in Norway. Most young stroke survivors are discharged from the health services within a few weeks or months after stroke. After discharge, the stroke survivors are difficult to identify, thus it was necessary to recruit participants by using a convenience sampling method [28]. The main strategy of recruitment was to place a notice on the website of the Norwegian Stroke Foundation inviting Norwegian speaking adults between the ages of 18 and 67 years who had suffered a stroke to participate in a qualitative interview study. Additionally, some stroke survivors were asked by acquaintances if they were willing to participate in the study. Although we used a convenience sampling strategy, we aimed to recruit a wide variety of participants with respect to age, gender, social status, and time since stroke onset. 

Recruitment continued until the information obtained in the interviews was determined to provide no new information. Of the 23 stroke survivors recruited for the study, three were recruited by acquaintances; the others responded to the announcement on the website. One participant did not sign the written consent form after two reminders and was therefore excluded from the study. 

### 3.2. The Participants

Twenty two participants (15 men and 7 women) aged between 20 and 61 years (mean age 44.8 years) who lived in urban and rural areas in the southern part of Norway gave their consent to participate in the study. The time since stroke onset ranged between six months and nine years. Despite the wide range of time since stroke onset, we determined all of these stroke survivors to be of interest, allowing us to obtain more detailed knowledge about living life with the aftereffects of a stroke during the semistable phase, which is defined as more than six months after stroke [29]. 

All but one of the participants were initially diagnosed with occlusion or haemorrhage of an artery in the left or right hemisphere of the brain. The one exception was diagnosed with a subarachnoid haemorrhage. When interviewed, most of the participants still suffered from a variety of symptoms such as upper and lower limb weakness/paresis, spasticity, cognitive impairments such as lapse of memory, concentration impairment, neglect, and aphasia. 

The participants were divided into three social groups, inspired by the Family Life Circle Framework [16] as follows: (1) young nonestablished participants, (2) participants living together with family, that is, from babies to teenagers, with or without a partner, and (3) participants without children at home, with or without a partner. 

Two of the participants (aged 20 and 27) in group one were students and single and were forced to discontinue their studies after stroke onset. When the interview took place, one of them tried to follow an education course without success, while the other one had not been able to return to the university to continue studies. One of the male participants, aged 25, had a girlfriend and had recommenced studies. A single male, aged 32, who was working prior to stroke onset, was on social security benefits four years after stroke onset. All but one of participants from the first social group lived on their own: the female participant lived with her original family. 

The ages of the participants in the second social group ranged from 32 to 61 years. Six were married, one was a cohabitant, one was divorced, and one was separated. The numbers of children living at home ranged from one to three, and they aged between 1 and 18 years. Among this group, three had returned to fulltime work, two were still on sick leave, and four were receiving social security benefits.

The oldest participant (aged 60) in group three (aged 48 to 60) was married and working part-time, one participant was working fulltime, and the other seven were receiving social security benefits. Three were single, two were divorced, and three were married. 

Some of the participants from groups two and three had grandchildren. For further details, see Table 1.

All the participants lived in their own homes. One of the participants in group two, and one in group three, needed assistance with activities of daily living. 

### 3.3. Data Collection Method

In-depth interviewing is an acknowledged method to explore experiences within qualitative research. The interviews gain access to participants' narratives, opening up the meaning of their lived world [26]. Thus, interviewing was the selected strategy to obtain data.

The interviews, carried out by the main author, lasted between 48 and 120 minutes and were conducted between January 2009 and April 2010. Twelve of the interviews took place in the stroke survivors' homes, seven in a learning and mastery centre, one in the stroke survivor's place of work, one in a café, and one in a hotel (for practical reasons). 

A thematic outline of open-ended questions was used to guide the interviews [27], focusing on the experiences of living a life after stroke. The experiences of what happened at the onset of stroke and experiences of the present situation were initially inquired about with thematic questions. Probing was used to encourage the participants to expand their responses related to the family context. 

### 3.4. Data Analysis Method

The hermeneutic phenomenological analysis was guided by Ricoeur's narrative interpretative theory [30] and conducted in three main interpretative steps. During the first step, all recorded and transcribed interviews were read several times to gain a preliminary “naïve” understanding of the content. The events and actions expressed during the interviews were then summarised into 22 “thumbnail portraits” that captured the plots of the participants' narratives [31]. A first interpretation of the experiences of the stroke survivor's family life was then formulated. During the second step, that is, the structural analysis, the different aspects of the experiences of the family situations expressed in the narratives were explicated by dividing the interview texts into meaning units and developing initial subthemes and themes. This expanded the interpretation of the plot line presented in the thumbnail portraits and facilitated a more thorough comprehension [30] of the experiences of a stroke survivor living within a family context. During the third step, the interpretation was further challenged, and the meaning was examined up against family theory. Accordingly, the interpretation was adjusted several times, and the texts were discussed, written, and rewritten, taking the context of the three different social groups into account. In the third step, the critical interpretation, the researchers further challenged the analysis and verified the most significant interpretations by comparing and contrasting the themes and subthemes in order to render a more comprehensive understanding of the family experiences of living after stroke for individuals between the ages of 18 and 67. The comprehensive understanding was verified by returning to the original narratives in order to check that the interpretation adequately reflected the experiences of the participants. Direct quotes from the narratives are used to illustrate the interpretation. 

### 3.5. Ethical Considerations

All participants were provided verbal information prior to being given written information about the study, and confidentiality was ensured. They were also informed that they could withdraw from the study at any time without consequences, as stated in the Helsinki Declaration [32]. The participants signed and returned an informed consent form to the researcher. After giving consent, the participants were contacted, and an appointment for the interview was scheduled. 

The project was approved by the Regional Medical Ethics Committee (REK) (Project number: 2.2007.37) and the Social Science Data Service in Norway (Project number: 16369). 

## 4. Results

The stroke survivors participating in this study experienced their present lives in light of their life prior to stroke onset and a longing for a meaningful life in the future. The plotline of the narratives highlighted that challenges within the family changed over time. From an initial struggle for normalcy and balance in the relationships within the family when returning home, the process shifted over time to a more resigned attitude. The challenges they experienced are summarised in two main themes: “struggling to reenter the family” and “screaming for acceptance.”

### 4.1. Struggling to Reenter the Family

This theme reflects the initial struggle to reestablish life as it was before stroke onset with respect to fulfilling family roles. It became obvious that the stroke influenced the participants' abilities to meet family expectations, and the analysis revealed that the participants struggled to reestablish their role identities, which is highlighted in the subtheme “facing the shortcomings.” The second subtheme, “the need for self-protection,” revealed that the stroke survivors need to protect themselves when facing their inabilities to participate sufficiently in family life and meet family demands. The situation called for an option of retreat to “survive” their new life of living with stroke. 

#### 4.1.1. Facing the Shortcomings

It was difficult for the participants to realise that they were less able to care for others, devote attention to others, or sufficiently engage in or carry out normal life tasks within the family. Facing this shortcoming influenced their identity, as they transformed from an independent, active, and participating individual to a person who is dependent on support from partners, children and/or parents:

*Yes, he [the cohabitant] is just amazing. What he does for me […] he is doing everything […] so incredible, that he does such things. But you feel yourself that it's not right. You really want to repay with something. It should be others coming here and do my part of the housework, so we could have some more time to be sweethearts. […] You get very dependent and simultaneously you don't want to be so dependent that others take your role. […] I think he is worn out (woman 38, six years after stroke).*

Parenting children or becoming parents close to the time of stroke onset brought even more challenges to the parenting role. Becoming parents is usually a positive life transition. However, parenting is a comprehensive activity that requires one to be able to fulfil myriad parental tasks that can be interrupted when one is unable to meet the expectations. A male aged 32 had his second child during the acute phase of the stroke. He expressed his struggle with this situation one and a half years after stroke:

*You are supposed to say that it was great [to have a new child] and it is. But others have told me that, “Now that you are at home, you can use your time to be together with your new son”. I couldn't do it. I've been at home for one year, but I've been absent-minded. […] So I didn't experience it [the birth of the new child] as something great at all. […] I would prefer another situation. I've stayed home for one year, but I haven't managed being there. […] It has cost her [my wife] something too, having a new-born and a two-year-old, that she has to practically care for alone. *

Emotional and cognitive changes such as being short-tempered, suffering memory loss, or experiencing slow thinking were expressed as challenges in parenting and the partner role. Situations that are normally considered manageable were described as difficult to overcome both among new and long-term stroke survivors. One of the fathers, aged 43, expressed the impact of a stroke on vulnerability nine years after stroke:

*At the beginning, with the kids, it was a problem that I was short-tempered. […] I got angry for nothing. I grew better [at managing it]. However, when this was under control, the teenage period started and new challenges arose. Then I was suddenly “too slow” and “I don't hear and I don't understand” [laughing]. […] I don't think I'm thinking fast enough. […] If I don't hear at once, forget it! […] It's difficult to differentiate how it would have been if I was healthy […] I've been talking to other parents of teenagers that say that it is just like that for them as well. So, on this point I'm not sure. We stroke sufferers are probably especially sensitive to these things.*

The established “behavioural standards” (i.e., the way things were generally carried out in the family) within the families continued, despite the fact that one of the family members is suffering from a stroke. The expectations of teenagers' participation in the household tasks did not seem to alter, although they were required to assist their parents with stroke. The single-parent families' vulnerability was apparent, as there was no other adult to share in the parenting responsibilities. Thus, the responsibility to care for children could result in a guilty conscience or in the sacrificing of the stroke survivor's personal needs:

*Well, it becomes a little too much sometimes. […] Now after she [the daughter aged 18] got pregnant and had a baby I feel that I don't manage to follow up the thirteen-year-old [daughter]. […] She [oldest daughter] is so young and […] needs help […] but I'm becoming exhausted […]. And then there are so many thoughts that wear me out (woman 38, four years after stroke).*

However, the established family roles could change as a result of the stroke survivor's inability to continue the former “executive roles” (i.e., carrying out the activities and responsibilities to solve designated tasks in the family). The involuntary role as a home worker was expressed as frustrating and uncomfortable for both women and men. In the narratives, the informants expressed that others were talking about how they just stayed at home and were not participating in society as expected. These worries were especially apparent among those stroke survivors struggling with invisible symptoms. 

Some of the participants had reconciled themselves to their new life following the stroke. They described their roles in staying at homes during the daytime as satisfactory. The contact and dialogues with family and friends contributed to making the situation tolerable. The years spent at home raising the children and being the principal home worker also had positive factors compared to being the traditional breadwinner. A nonbusy life offered the stroke survivor the opportunity to make new choices that placed greater emphasis on family life and home care combined with the possibility to take better care of the children. One father whose children were aged two to five years at stroke onset stated the following:

*[To be at home] has been positive. […] Not all fathers have that opportunity. I've had the opportunity to be there for my kids, pick them up at school and make dinner. […] That's somehow the great, positive part of this (male 43, nine years after stroke). *

#### 4.1.2. The Need for Self-Protection

Although a busy family life still demanded participation, fulfilling the expected roles was hard to manage and called for a retreat from participating in activities inside and outside the family. The suffering associated with not being able to fulfil the family role was expressed as a bodily pain:

*A lot has happened to you. […] Emotionally, if I get into situations where I discuss or if there is a quarrel or discussion or something like that, I often don't manage to talk at all. […] I burst into tears, and that can happen both when I'm sad and happy. […] It's not a normal cry, […] but it is like something cracks inside you. And you lose much of your self-control. […]. It is difficult. […] Because when she [wife] is irritated, she feels that I utilize the crying and the emotions as a means for sympathy. […] She says my behaviour is different. That I'm slower, that I don't perceive [or] hear as well as before. I'm not present like before. […] [It is hard] because I don't wish to defend myself, I don't wish to have changed. I wish to be the one I was. I wish to be myself. And that she experiences this, is sad (man, 50, three and a half years after stroke).*

Withdrawing seemed the best way to “survive” and avoid the questions when living alone. The single participants talked about their choices with respect to staying alone, being able to avoid questions from others, and being able to protect themselves from situations that may negatively influence their self-esteem. Being single and independent resulted in fewer commitments to fulfil family roles or student roles. Accordingly, these participants were able to isolate themselves from having to give reasons for not participating. One male participant stated: “You get some warning to not immure yourself, correct? I think of that sometimes, that I'm there. That I'm not out on something. So […] I've something to do there” (man, 49, three and a half year after stroke).

The participants with adult children also discussed the opportunity to withdraw from participating in extended family activities. Being forced into a self-protective role was experienced as doubly cumbersome. On the one hand, the survivors felt vulnerable in situations where they could not fully participate as desired, while, on the other hand, they were frustrated because they struggled to participate. Not being able to fulfil the role of grandparent was expressed as a sorrow and a loss. Accordingly, the struggle of not being able to properly relieve their grandchildren's parents was expressed as a shame:

*I don't think I've been lucky. I have two grandchildren aged five and three. I'd imagined having the grandchildren here. […] But after two hours, I'm completely exhausted because [the oldest] talks all the time. […] So I am not able to be what I wished to be, a real grandma. That is terrible. […] And you would like to be a mum, you should not be a suffering patient either. […] I have at least tried to explain without too much complaining that I don't manage being a babysitter the way I wished, and I can't be grandma the way I wish. But, as they say, […] you are the grandma you can be, and that's enough [crying] (woman, 55, two and a half years after stroke).*

However, being together with grandchildren was perceived as a meaningful experience when nonbinding and sociable. 

The single nonworking or nonstudying participants stayed in their homes more than desirable and described their days as boring. They missed their friends and ruminated about their absence, while simultaneously expressing an understanding that their friends and associates were occupied with their own day-to-day-activities. However, one of the single participants, who had recently found a girlfriend some months prior, viewed his situation as satisfying. He explained that while his social contacts had changed, it was a conscious choice. He had made a decision to prioritise focusing on his work during the week-days and being with his girlfriend during the week-ends in the period before his girlfriend moved in with him. 

The stroke survivors in parenting roles expressed that their day-to-day challenges at home demanded their presence. They viewed their lack of extended social relationships as temporary because their current tasks demanded that they care for their children. They expressed less ability to choose a self-protecting life style because of their own and their families' expectations that they participate and engage actively in family life. Accordingly, they felt they were unable to find the leisure time necessary to recover. They described their lives as busy, which is opposite to that of those stroke survivors not living in an immediate family. The family expected attention following a situation filled with challenges and commitments. Although they felt shame as they struggled for personal “self-protective time,” they still tried to schedule their own activities such as sports activities, walking the dog, or hunting:

*It has to do with recovering, to make sure to go out and do things that give me positive energy. I find that important. And to be good at thinking “this is my need, this is for me” (woman, 46, seven months after stroke).*

### 4.2. Screaming for Acceptance

When recovery was delayed, the interest and support from others changed, becoming more silent and influencing life in a more negative manner. The stroke survivors progressed from being treated with “silk gloves” and being the “commander” close to stroke onset to a more secluded situation. The support offered from others gradually decreased and changed, as expressed in the subtheme, “the silent cry for acceptance.” Close to stroke onset, the dialogues focused on the stroke survivor's feelings of living with stroke, and, later on, the communication altered to be more oriented on practical tasks to fulfil daily family roles. The attention from others decreased, though the suffering from the illness still remained a day-to-day challenge. The second subtheme in this section, “living on charity,” reflects the stroke survivor's insecurity about how to “survive” as a family-member when living a life that is marked by chronic consequences as the result of a stroke. 

#### 4.2.1. The Silent Cry for Acceptance

The stroke survivors thought about life following their strokes and the slow recuperation process despite being “young”, thinking that a stroke was an illness that only affected the elderly and retired. Involvement from the immediate and extended family increasingly decreased as time passed. The stroke survivors expressed a decreasing engagement from others as a barrier from discussing their situations. They presumed that others did not understand, and, therefore, they avoided bothering others:

*[…] She [wife] works in an institution. I'm not sure which, but the patients there have different [problems], are both old and young, and she has folks there who had have the same injury as me, and are suffering worse. I could have run the risk of being in an institution myself. So [my current problems] are really nothing. […] We only talk about the epilepsy, the other things are not a theme any longer. Talking about it [the stroke] is finished (man, 38, four and a half years post-stroke). *

The participants expressed frustration about being misunderstood by people around them. They suspected that others noticed their disabilities, but they never inquired about their conditions. Because of this lack of attention from others, they tried to live life as normally as possible.

The narratives expressed a sense of discouragement about the lack of understanding from friends, even though the relationships were close. The stroke survivors wanted others to understand their present situation and their inability to participate in activities and gatherings the way they had before their stroke:

*I don't join them on a night out […] I [am not able] to dance […], I am not active the same way they are, [it would] not be very social, so it is no point (woman, 20, one year post-stroke).*

Some of the nonestablished stroke survivors expressed difficulty telling their parents about their illness, as they preferred to remain independent and protect their parents from worrying about them, thus avoiding being overprotected:

*I don't want to reel off all my health problems to everyone […] I feel it's my problems. […] I don't want to trouble the family. […] So I probably try to protect them [from getting worried]. […] They [the parents] don't know more than others. And I think that's okay. I didn't mention when I went on sick leave or when I was on vocational rehabilitation. My mother would have been scared and start to nag at me, call me every day and ask for my condition […] I don't manage that nagging. […] I've a mother that worries a lot […]. So it's better to [not tell them] […]. But I don't know how she's thinking about it (man, 32, four years post-stroke).*

The longing for sympathy experienced at stroke onset was also highlighted, and the suspicion from others of exaggerating the challenges that some of the stroke survivors expressed was difficult to overcome. Nevertheless, some of the informants expressed the dialogue as positive. The stroke had resulted in paying more attention and becoming closer to spouses, children, parents, and adult siblings:

*I'm feeling good […] I want to be together with my family, relax and do things I neglected when I was working “twenty-four hours a day”. […] Me and my wife […] were forced to talk about it [the stroke]. […] The siblings are thinking of me. [We are] touching things we didn't before. […] After all, the relations are stronger (male, 49, three years post-stroke).*

#### 4.2.2. Living on Charity

As life continued, the stroke survivors continued to struggle with fulfilling family roles and obligations, struggled with balancing protection and participation, and struggled with telling others to gain an understanding of what it meant to live with stroke. Life after stroke was compared to living in a box or in a shell, struggling to be released and to become more independent. The participants expressed a kind of resignation about telling their partners, children, friends, parents, and grandparents what living with stroke was like. They put a lid on the situation, continuing to suffer from illness. This led to a state of defencelessness. One of the participants aged 52 was nevertheless attentive to how his stroke influenced his wife six months after stroke onset. He tried to ask her specific questions and encouraged her to tell him her struggle to maintain the open dialogue that was important to the family. Even if the stroke survivors were judging that they could manage more participation, they strived to communicate that they possibly could be more responsible in their roles:

*The problem is that I don't have sensibility in one of my hands. That is difficult, when I'm going to change nappies, dress [the child] and such. Sometimes I can hurt [the child]. It has happened that I have pinched. It's difficult. […] I think she [the wife] has done too much. […] It looks like she wants to, but I still think, it's a kind of naturally, that the mother becomes the main [parent] the first years. […] I think I can do more, but […] it has to be good for the kid (male, 38, four and a half year post-stroke).*

Some of the stroke survivors expressed a fear of being rejected by the family and left alone. They ruminated about a prospective scene where the family would become fed up with the stroke survivor's dependence and failure to fulfil their expected role and envisioned how the family would go on once the children had grown up and moved outside the home. Following this negative thinking, the strategy of the stroke survivor was to not complain and hope the family could persevere with a nonfunctioning family member:

*The worst thing to think about is if they [my family] become fed up with me and throw me out. […] Therefore, I have to try to remain as cheerful as possible. […] Even worse is if someone finds out that they don't want to have anything to do with me. […] Sometimes this thinking comes to me on [difficult] days […] (man, 61, four years post-stroke). *

Two of the single stroke survivors expressed regret that their intimate relationships came to an end shortly after stroke onset. They expressed the desire to establish contact with new friends and develop intimate relationships. Although they did not understand why some friends were not visiting at all, they interpreted their lack of presence as a difficulty in being around ill people. They understood the fact that their friends gave up on the relationship to focus on their personal life projects, and the stroke survivors had to realise that their own lives were progressing in a different way:

*I've lost a lot of friends. Many friends think of me, but they don't get in touch. I always have to call them first, sure because of the injuries. […]. When I contacted them the first time, I also have to contact them the second time […]. When they come back [to hometown] with their kids and families […] they have almost forgotten me. […] We chatt on the phone, but if I visit them, it becomes only that time. […] I miss school. […] If I start at a new school, I'll make some more friends. […], establish a new social network and get a job afterwards (man, 27, three years post-stroke).*

Stroke survivors who were in the lower socioeconomic strata encountered increased challenges with respect to participating in family activities. Living on disability benefits prevented some of the participants from participating in social events. Other participants explained that they prioritised staying at home to save money and pay for their children's leisure activities. Some of the nonestablished stroke survivors wanted to become financially independent so they could live by themselves and be not dependent on family support. 

Regardless of the disabilities incurred following a stroke, none of the stroke survivors gave up, and they continued to hope they would be able to fulfil their roles and participate in meaningful activities. Additionally, hoping to find a partner, one's complete education or establishing a family were also important to some of the stroke survivors. 

## 5. Discussion

This study highlighted that maintaining roles and interactions within the family as a working-age stroke survivor was challenging, as summarised in the two main themes “struggling to reenter the family” close to stroke onset and “screaming for acceptance” later on in the stroke trajectory. Although the participants' experiences as stroke survivors ranged from six months to nine years, their descriptions regarding life since stroke onset confirmed some common experiences. As time passed, even though it was expected that life would become normalised, it remained difficult for stroke survivors to fulfil certain roles and to interact within and outside the family many years after stroke onset. 

All but one of the nonestablished participants in this study were dissatisfied with their current lives. From being left by their partners or significant others to being forced to discontinue their studies or their work and finding it difficult to maintain friendships changed the lives of the participants and isolated them from living a normalised life. The participant who found life different, but was still satisfied, had established an intimate relationship, and succeeded in being independent and emancipated by resuming studies and work was perceived to be of high value to young adults [16]. The need for emancipation was also expressed by their desire to live with minimal intervention from their families. This differs from what was found by Teasell et al. [19] who found that younger stroke survivors had a tendency to increase their dependency on their parents. The struggle to maintain relationships and intimate relationships is also evident elsewhere [1, 2, 21].

Stroke survivors in parental roles did not express the same opportunities to isolate themselves from activities as did the nonestablished group. In fact, they expressed the opposite, as their lives were busier because of parenthood commitments and demands within the family. The inherent responsibilities of being a parent [16] could result in a conflict between managing time for parental tasks while simultaneously finding time to focus on recovering from their stroke. The expressed struggle with raising children aged one to eighteen years emphasised the fact that the upbringing of children is comprehensive [16, 18], making the situation even more challenging for stroke survivors when the flexibility that is necessary to interact with children [16] may be interrupted by psychological challenges following the stroke. While mothering is found to be challenging when suffering from illness [1, 9], as this study confirmed, the fathers also worried about their inability to fulfil their parental roles. The fathers' expressed emotional shortcomings in their parental roles go beyond their need to protect their families both physically and economically, as noted by others [1, 12, 20]. 

It is important to underline that although participants in parental roles struggled to fulfil that role, some fathers expressed that they could have contributed to the family life more than they did. However, because of poor communication regarding how to participate, some felt that they were forced to withdraw from fulfilling their parent roles as expected. Other fathers, however, found a new role as the principal parent who stayed at home. This highlights the need for health services to be aware of the general vulnerability of stroke survivors in parental roles, and to be particularly aware of the fathers' struggles to fulfil their roles. 

The participants without children and those with children that had moved out of the home also perceived their lives as challenging because of the remaining psychological symptoms. Their family challenges were more specifically related to their role as grandparents, a point not highlighted in previous studies, and to a struggle to maintain contact with friends. Furthermore, couples in long-lasting marriages have greater opportunities to create a new marriage path, even after the partner has suffered a stroke [9, 11].

The different life situations indicate that living with stroke is not easy for any of the social groups analysed here. The expressions of life with respect to facing struggles of not being able to fulfil role expectations resulted in negative mental activities. Although the support from family is important [9], nonestablished stroke survivors as well as stroke survivors living with children seem to be particularly vulnerable because of the stroke's impact on life at a time when one is expected to be emancipated from the family of origin. These findings confirm adults with families and children as vulnerable after stroke and in need of support in later phases of recovery [33]. In addition, access to practical childcare would be helpful for those stroke survivors with young children.

### 5.1. Strengths and Limitations

The participants in this study were recruited using a convenience sampling method, due to the difficulties getting access to this subpopulation of stroke survivors. Although this is a less preferred method for qualitative research than maximum variation sampling [28], the applied sampling strategy generated a wide variety of participants with respect to age, gender, social status, and time since stroke onset and therefore the participants represent a broad range of experiences. Furthermore, they provided rich and detailed descriptions of their experiences and concerns. Accordingly, we maintain that the findings provide in-depth knowledge regarding family life challenges during the recovery phase ranging from six months to nine years. 

Despite the participants' variations in time since stroke onset, the narratives expressed significant commonalities across age and social situation. These findings, together with the differences across the family cycle, underscore the challenges faced by long-term stroke survivors and the need for better followup. However, maximum variation sampling would have strengthened the transferability [28] of the findings. 

Validation of the findings was sought through the research group's continuing and open discussions during the analysis process until consensus about the findings was attained. 

## 6. Conclusion

Being a stroke survivor between the ages of 18 and 67 poses significant and multifaceted challenges to family life many years after stroke. To interpret and manage the family situation calls for support across the life span, suggesting that individual followup by health services would be helpful. Nonestablished people living with stroke seem to be particularly vulnerable, as they are often isolated in their homes. It is also important to be aware of the vulnerability of stroke survivors as they attempt to fulfil their parental roles as both mothers and fathers. The daily demands and their struggles to attend to family life leave them in need of tailored followup with regard to how to arrange their daily demands of childcare and marital relations. Being provided opportunities to narrate their experiences outside the home context might be helpful to prevent psychosocial problems. 

## Figures and Tables

**Table 1a:** (a) Social group 1: Young non-established participants

Sex	Age	Consequences of stroke	Time since stroke onset	Civil status	No. of children at home
Male	32	Subarachnoid hemorrhage. Concentration and memory affected.	4 years	Single	0
Male	27	Thrombosis left hemisphere. Hemiplegia right side. Aphasia. Concentration and memory affected.	3 years	Single	0
Male	25	Hemorrhage cerebellum. Slight visually impaired.	4 years	Single	0
Female	20	Thrombosis right hemisphere. Hemiplegia left side.	1 year	Single	0

4 of 22 (1 woman, 3 men).

**Table 1b:** (b) Social group 2: Participants living together with family, that is, from babies to teenagers, with or without a partner

Sex	Age	Consequences of stroke	Time since stroke onset	Civil status	Age of children at home
Female	46	Emboli right hemisphere. Visually impaired. Aphasia. Concentration and memory affected.	7 months	Separated	6 and 11 years
Male	61	Hemorrhage right hemisphere. Hemiplegia left side.	4 years	Married	16 years
Female	38	Hemorrhage right hemisphere. Concentration and memory affected.	4 years	Divorced	13 and 18 years
Female	38	Thrombosis left hemisphere. Visually impaired. Apraxia. Aphasia. Concentration and memory affected.	6 years	Cohabitant	2 years
Male	32	Emboli right hemisphere and cerebellum. Concentration and memory affected.	1,5 year	Married	1 and 3 years
Male	50	Thrombosis right hemisphere. Aphasia.	3,5 years	Married	13 and 18 years
Male	43	Thrombosis right hemisphere. Hemiplegia left side. Memory affected. Epilepsy.	9 years	Married	12, 14 and 15 years
Male	38	Hemorrhage right hemisphere. Hemiplegia left side. Epilepsy	4,5 years	Married	4 years
Male	52	Thrombosis cerebellum. Numbness right hand.	6 months	Married	14 and 16 years

9 of 22 (3 women, 6 men).

**Table 1c:** (c) Social group 3: Participants without children at home, with or without a partner

Sex	Age	Consequences of stroke	Time since stroke onset	Civil status	No. of children at home
Male	49	Hemorrhage cerebellum. Reduced strength left side. Epilepsy	3 years	Married	0^1^
Male	54	Hemorrhage right hemisphere. Concentration and memory affected.	1,5 year	Married	0^1^
Female	55	Thrombosis left hemisphere. Concentration affected.	2,5 years	Divorced	0
Male	60	Thrombosis right hemisphere. Hemiplegia left side. Neglect. Concentration affected.	4,5 years	Married	0
Female	48	Hemorrhage right hemisphere. Hemiplegia left side.	6 years	Single	0
Male	54	Not specified.	8 years	Single	0
Male	56	Hemorrhage right hemisphere. Concentration and memory affected.	9 years	Married	0
Female	58	Thrombosis right and left hemisphere. Neglect. Concentration affected.	1,5 year	Divorced	0
Male	49	Thrombosis right hemisphere.	3,5 year	Single	0

9 of 22 (3 women, 6 men).

^1^This participant had an adult son who lived at home.
